# Artificial intelligence in arthroplasty

**DOI:** 10.1186/s42836-021-00095-3

**Published:** 2021-11-02

**Authors:** Glen Purnomo, Seng-Jin Yeo, Ming Han Lincoln Liow

**Affiliations:** 1 St. Vincentius a Paulo Catholic Hospital, Surabaya, Indonesia; 2 Adult Reconstruction Service, Department of Orthopaedic Surgery, Singapore General Hospital, Singapore, Singapore; 3 Duke-NUS Medical School, Singapore, Singapore

**Keywords:** Artificial intelligence, Deep learning, Machine learning, Arthroplasty, Joint replacement

## Abstract

Artificial intelligence (AI) is altering the world of medicine. Given the rapid advances in technology, computers are now able to learn and improve, imitating humanoid cognitive function. AI applications currently exist in various medical specialties, some of which are already in clinical use. This review presents the potential uses and limitations of AI in arthroplasty to provide a better understanding of the existing technology and future direction of this field.

Recent literature demonstrates that the utilization of AI in the field of arthroplasty has the potential to improve patient care through better diagnosis, screening, planning, monitoring, and prediction. The implementation of AI technology will enable arthroplasty surgeons to provide patient-specific management in clinical decision making, preoperative health optimization, resource allocation, decision support, and early intervention. While this technology presents a variety of exciting opportunities, it also has several limitations and challenges that need to be overcome to ensure its safety and effectiveness.

## Background

Artificial Intelligence (AI) is a subfield of computer science that uses computers to emulate human cognitive functions and carry out tasks that can match or exceed human performance [1, 2]. Traditionally, specific computer codes were required to explicitly instruct machines how to process data and make decisions. Through Machine Learning (ML) algorithms, computers can learn and improve from experience without exact instructions by using large sets of data inputs and outputs to recognize repetitive patterns. This subset of AI emphasizes the learning aspect of computer intelligence to create autonomous resolutions [1, 3, 4].

Deep Learning (DL) is a more advanced and complex form of ML that mimics the neuronal connections of the brain by creating an Artificial Neural Network (ANN) [1, 2]. This algorithm can learn from unstructured and unlabeled inputs without supervision and segregate data input from low relevance variables for the prediction of interest. Deep Neural Network (DNN), which contains multiple hierarchical levels of ANN, is required to improve data predictions and allow the development of models without explicitly programmed directions [1–3]. Convolutional Neural Network (CNN) is the other form of DL which is used for computer vision tasks including medical image analysis [5].

The application of AI has expanded prominently in the medical field due to advances in computing power, learning algorithms, data storage, and the availability of large-high-quality data sourced from electronic medical records and wearable health trackers [1, 2]. Although its adoption is still in early phases, AI has been extensively used across many fields in medicine such as radiology [6, 7], cardiology [8–11], dermatology [12–15], ophthalmology [16, 17], neurology [18, 19], oncology [20, 21], gastroenterology [22, 23], and respiratory medicine [24]. Some examples of clinical applications that have been approved by the US Food and Drug Administration (FDA) include *Arterys* for cardiac magnetic resonance image analysis, *Idx* for detection of diabetic retinopathy, and *MammoScreen* for breast cancer screening [25, 26]. In fact, the number of AI/ML-based medical devices approved by the FDA and *Conformité Européenne* (CE) mark in Europe increased significantly from 9 and 13 in 2015 to 77 and 100 in 2019, respectively [26].

In the field of orthopedics, the influence of AI has also grown rapidly for the past two decades [4]. Currently, this technology has the potential to be utilized in various areas such as image-recognition diagnostics, surgical risk prediction, generating patient-specific payment models, augmenting clinical decision making, and outcome prediction [2]. Thus, AI enables surgeons to select the ideal patient for the best surgical outcomes, develop a patient-specific surgical plan, and prepare for patients who are at high risk of complications [27].

This article reviews the current applications of AI in the field of arthroplasty and the evidence supporting its utilization in the clinical setting.

### Diagnostic tool

Computer-aided diagnosis could be utilized to help physicians make decisions because of its objective nature and a high degree of accuracy. CNN has demonstrated breakthroughs in a variety of general image recognition applications. The accuracy of these applications is at near-human levels and has the potential to outperform human experts in the future [28]. These models can be used to aid physicians to diagnose osteoarthritis, assess the severity of osteoarthritis, or even detect osteoarthritis in presymptomatic individuals and predict the need for joint arthroplasty [28–34].

Diagnosis and progression of osteoarthritis (OA) based on knee radiograph can be made by CNN. Several algorithms have been proposed to detect knee OA based on knee radiographs. By using plain radiographs from the OsteoArthritis Initiative database, Brahim *et al* demonstrated the ability of their method to detect OA, even at an early stage [35]. Another DL model developed by Xue *et al* was able to generate a diagnosis of OA automatically using a radiograph without human involvement. This algorithm could accurately identify radiograph features that are associated with hip OA such as joint space narrowing and osteophytes, allowing it to function like a radiologist with 10 years of experience [29]. Both Antony *et al* and Tiulpin *et al* presented algorithms that were able to automatically diagnose and classify the severity of knee OA from plain radiographs [28, 32]. Most recently, the model proposed by Leung *et al* not only detected knee OA but also predicted the development of OA which led to eventual total knee arthroplasty (TKA) within 9 years [31].

In a different application, early diagnosis of OA in presymptomatic individuals may allow initiation of disease-modifying therapies to modify the course of the disease. Kundu *et al* demonstrated the use of AI to detect OA 3 years before the individuals became symptomatic. They used 3D Transport-Based Morphometry to identify water distribution patterns in cartilage tissue that is captured by MRI [30]. In addition, Hirvasniemi *et al* used ML to predict incidents of radiographic hip OA or TKA over 10 years. They utilized bone texture analysis of proximal femur and acetabulum from plain pelvic radiographs to predict incidents of radiographic hip OA [33].

The recent technological advances in AI have proven that image recognition applications can detect prosthetic loosening. This is particularly useful as aseptic implant loosening remains one of the main causes of failure in arthroplasty. Shah *et al* showed that loosening of implants could be detected on plain radiographs by ML with an accuracy of 95.6%. This progress could assist surgeons in diagnosing the condition without the need for expensive imaging modalities such as fluorodeoxyglucose-positron emission tomography (FDG-PET scans), bone scans, magnetic resonance imaging (MRI), and arthrograms, which have not been demonstrated to significantly improve diagnostic accuracy [36].

### Patient-specific payment

The use of AI to preoperatively predict the length of stay in the hospital (LOS) and payments for patients undergoing arthroplasty based on patient-specific factors can help optimize value-based care and enable the development of a patient-specific pricing benchmark for governments and insurance companies [37–39]. By accurately predicting the LOS of patients, decision making and inpatient bed assignment can be simplified so that medical resources can be allocated to the maximum [40]. Doctors can also develop recovery plans quickly and provide better services as they can adjust patient expectations and provide early interventions [38]. High-cost predictions allow hospitals to channel additional resources to at-risk patients, to prevent anticipated complications and reduce overall costs [37].

Several studies have demonstrated the ability of ML to predict LOS and hospitalization costs for both TKA and total hip arthroplasty (THA) [37, 39, 41, 42]. ML is proven to provide predictions with fair to excellent construct validity, reliability, and responsiveness before primary TKA and THA [39, 41, 42].

Li *et al* conducted a study to compare the accuracy of the ML model with two logistic regression models in predicting LOS of patients undergoing primary unilateral TKA. They found that the ML algorithm had better accuracy in predicting LOS [38]. Another similar study has also demonstrated better predictive performance of their ML models compared to conventional methods, including logistic regression [40].

### Preoperative evaluation

AI can be used during preoperative screening to identify high-risk patients thereby allowing doctors to prepare preventive measures. Jo *et al* conducted the first study on the use of ML to predict the transfusion risk after TKA while Karhade *et al* performed the first investigation into the use of ML in predicting the risk of having prolonged postoperative opioid prescriptions in patients undergoing THA. Both studies have been validated and showed good performance [43, 44].

Novel use of AI has been proposed for the preoperative evaluation of revision arthroplasty. While accurate identification of the manufacturer and type of implant is required for preoperative planning of revision arthroplasty, it is estimated that surgeons are unable to recognize the implant preoperatively and intraoperatively in about 10 and 2% of cases, respectively. Inability to identify implants can lead to unpreparedness which may contribute to increased surgical time, perioperative morbidity, and overall healthcare costs [45–47]. Deep learning has been shown its ability to recognize implant manufacturer and design in hip and knee arthroplasty [45–49].

A recent study by Karnuta *et al* showed that their ML algorithm was able to distinguish between 9 unique knee arthroplasty implants including TKA, Unicondylar Knee Arthroplasty, and Distal Femoral Replacement from four leading manufacturers with 99% accuracy simply by evaluating anteroposterior (AP) radiographs [46]. In hip arthroplasty, deep learning algorithms are also capable of recognizing THA implants based on AP radiograph. Although the classification is based solely on identifying the design characteristics of the femoral stem, studies have found the results to be very accurate, even reaching an accuracy rate of 99.6 to 100% [45, 47, 49, 50].

Jodeiri *et al* estimated Pelvic Sagittal Inclination (PSI) from preoperative plain radiographs to augment the position of the acetabular component, eliminating the need for a Computed Tomography (CT) scan. PSI is increasingly being recognized to play a crucial role in acetabular component positioning as the optimal anteversion and inclination angle of the acetabular cup can reduce the risk of dislocation or impingement after THA. This study demonstrated encouraging results, with an accuracy rate of 80%. Future development of this model will enable the recognition of individual dynamic changes of PSI to enable patient-specific placement of the acetabular component in patients undergoing THA [51].

The application of these novelties during preoperative evaluation has the potential to assist surgeons in making clinical decisions, providing patient-specific planning, and improving outcomes.

### Outcome prediction

The potential ability of ML to predict patient outcomes after arthroplasty has been demonstrated by several authors [52–56]. Prediction of outcomes may facilitate the shared decision-making process between orthopedic surgeons and patients, particularly to decide whether the procedures will meet the patient’s expectations [53–56]. Furthermore, early identification of patients at risk of not having significant changes in postoperative Patient-Reported Outcome Measures (PROMs) may necessitate closer postoperative patient follow-up and optimize decision support before surgery [52].

Several studies focused on achieving minimal clinically important difference (MCID) after surgery, which refers to the improvement of PROMs necessary for a patient to consider the intervention beneficial or meaningful. Supervised machine learning algorithms developed by Fontana *et al* were shown to have fair to good ability to predict 2-year postsurgical MCID for general and joint-specific health PROMs [52]. Recent studies by Kunze *et al* and Harris *et al* also found that ML was able to predict the MCID of patients undergoing THA and TKA, respectively, so that it could help in optimizing preoperative health, improving patient selection, education, and satisfaction [55, 56].

Another outcome measure used to evaluate the efficacy and value of the intervention is postoperative patient satisfaction [54, 57]. Farooq *et al* found that compared to statistical models, ML algorithm had greater accuracy in predicting satisfaction after TKA [57]. Another study conducted by Kunze *et al* in patients undergoing TKA also showed that ML had good discriminatory capacity and superiority over standard logistic regression to identify patients at greatest risk for dissatisfaction. Accurate prediction of patient dissatisfaction following primary TKA may allow for preoperative health optimization and improved patient-doctor discussions [54].

Shohat *et al*. used ML to assist in treatment decision making of acute prosthetic joint infection (PJI). Debridement, antibiotics, and implant retention (DAIR) procedures have low morbidity with unpredictable results and variable failure rates. The authors developed an algorithm that could accurately predict the success of DAIR based on patient’s clinical presentations, comorbidities, physical examination, and laboratory results [58].

Shat *et al* did a retrospective cohort study of 89,986 patients undergoing primary THA. They developed an ML algorithm and compared their model with logistic regression and standard benchmark ML models in predicting major complications after THA, including infection, venous thromboembolism, cardiac complication, and pulmonary complication. Their model showed superior risk prediction compared to logistic regression. The accurate prognostic information obtained by this algorithm may facilitate decision making before surgery and augment postoperative clinical care [59].

### Postoperative evaluation and monitoring

Newer technology targeted at improving rehabilitation following THR and TKR provides a more objective outcome measure as compared to traditional patient-reported activity questionnaires. Wearable devices can record the movement profile of patients postoperatively, which can be used to assess their functional recovery [60, 61]. Continuous data from the device can be analyzed by ML algorithms which can lead to reliable measurement of functional outcomes after arthroplasty [62].

Ramkumar *et al* analyzed perioperative data collected by patient smartphones and wearable knee devices of patients undergoing TKA. Analysis of the data taken pre- and post-surgery including mobility (steps per day), range of motion (maximum knee flexion), home exercise compliance, opioid consumption, and PROMs, demonstrated that the remote patient monitoring enabled the authors to evaluate the mobility and rehabilitation compliance of patients after TKA. By analyzing data collected through advances in technology with ML algorithms, they proposed that these applications can help surgeons to identify potential causes of unfavorable outcomes [62].

Polus *et al* did a prospective study investigating the recovery of 72 patients undergoing primary THA for end-stage OA. All patients were instrumented with a wearable sensor system. This study showed that ML could predict the fall risk in post-THA patients by collecting objective functional data using wearable devices. They succeeded in predicting the risk of fall at 6 weeks after surgery with a high level of accuracy. By grouping high and low fall risk patients, fall prevention measures can be enhanced for the high-risk group, whilst an accelerated recovery program can be implemented for the low-risk group [63].

As another example, Rouzrokh *et al* used DL to automatically measure the acetabular component angles on postoperative radiographs. After building their algorithm based on two cohorts of 600 AP pelvis and 600 cross-table lateral hip postoperative radiographs, they found that their model was highly accurate so that it can be utilized not only in research settings but also in clinical settings [64].

### AI for surgical robotics

Robotic-assisted surgery enhances the surgeon's ability to perform more precise and accurate procedures more consistently with more patient-specific plans [65]. Many surgical robotic platforms have emerged in the last few decades. Despite progress, current robotic platforms are incapable of performing autonomous tasks and making cognitive decisions similar to those of humans [66]. AI may improve the ability of the surgical robotic system to perceive complex *in* *vivo* environments, conduct decision making, predict, and perform desired tasks with increased precision, safety, and efficiency, either under or without supervision from human control [67, 68].

The application of AI in robotic surgeries may reduce human errors and operative times [69]. Future surgical robots are expected to be able to apprehend and understand complex environments, perform real-time decision making, and perform desired tasks with increased precision, safety, and efficiency [68]. Recent study by Li *et al* used AI for the first time to guide the 3D reconstruction of CT data of lower limbs for facilitating robotic-assisted TKA. They used CT data of 200 lower limbs for AI-based 3D model construction and CT data of 20 lower limbs for verification. The result showed that the performance of AI-guided 3D reconstruction for robotic-assisted TKA was similar to that of the operator-based approach [70].

### Clinical decision making and future directions

Advances in technology and the use of AI provide opportunities to provide data-driven, high-performance medicine that can rapidly improve the field of arthroplasty [71]. Leveraging its potential to handle and optimize highly complex datasets, a future where the positive impact of this technology in healthcare is already visible [72]. Due to the ability to process large amounts of complex data to guide and predict outcomes, AI platforms have the potential to provide decision support to doctors, patients, and insurance companies [27, 72].

Arthroplasty surgeons will be able to select ideal patients for surgery, create patient-specific surgical plans, predict clinical outcomes and implant survival, and identify patients at high risk of complications [27]. In addition, the potential of AI to forecast treatment episodes offers unique predictive possibilities for generating tiered bundle pay models specific to patient complexity before arthroplasty procedures, enabling fair arbitration between surgeons, hospitals, and insurance companies [41]. AI’s ability to predict outcomes will facilitate arthroplasty surgeons in discussing possible surgery outcomes, making optimal joint decisions with patients before surgery, and prioritizing resources for postoperative monitoring [52].

Advanced algorithms offer an avenue to learn and adapt to different datasets including those relating to a patient's physical and psychosocial health and well-being from different populations and practices. Highly sophisticated analysis of a wide range of data is capable of generating impactful metrics that can be used to aid in better decision-making processes [73]. Jayakumar *et al* conducted a randomized clinical trial of 129 patients with knee pain associated with OA. They demonstrated statistically significant improvement in decision quality, level of shared decision making, patient satisfaction, and functional outcomes in patients using an AI-enabled decision aid. The results of this study suggest that AI-enabled patient decision assistance can provide a personalized, data-driven approach and improve shared decision making in the management of knee osteoarthritis [74].

Given the rapid technological advances, the widespread application of AI in the field of arthroplasty is expected to provide personalized health care by improving diagnosis, clinical decision making, patient care, and outcomes for specific patients.

### AI limitations and challenges

Apart from various interesting and seemingly promising applications, AI also has its limitations. The development of ML algorithms requires large amounts of data. ML developed using data from one setting cannot be used immediately by other practice settings in other locations because the training data may not representative of the population [75]. Algorithms with non-generalized data can lead to bias, possibly providing inaccurate recommendations for minority subgroups for which training data are less inclusive [76, 77]. To prevent algorithmic bias, ML should be designed according to the global community. In addition, clinical validation must be carried out using a representative population of the area where this algorithm will be used [77].

Additional local training data may be required for algorithm adaptation in new populations [75, 77]. Hospitals or clinics with too little data will face problems training the algorithm optimally so that sharing data with each other is necessary to achieve successful adoption of AI in healthcare at scale [76]. In data sharing, privacy and data protection issues can be a problem. AI developers must protect personal information and any other information beyond the use of a doctor-patient relationship that may harm the patient, such as the impact on health or other insurance premiums, job opportunities, or even personal relationships [76]. Moreover, there are also unique challenges and risks associated with cyber security threats [76, 78].

Transparency and trust are other issues. A lack of transparency in AI makes accountability and liability problematic [76, 79]. Some ML algorithms have a black-box phenomenon in which a logical explanation of how the output is generated is unknown [80]. The inability to explain why and how an algorithm derives certain decisions makes implementing AI difficult [77]. Explainable and interpretable algorithms are necessary not only to detect biases but also to facilitate transparent and trustworthy AI systems [76, 77].

## Conclusion

The adoption of AI in healthcare is inevitable. Currently, many studies are demonstrating the use of AI in various fields of arthroplasty. The application of ML in clinical practice will allow physicians to improve clinical decision making, anticipate problems, allocate resources, and provide personalized early intervention for each patient. AI has the potential to increase surgeon effectiveness and reduce human errors. Shortly, this technology will surely help arthroplasty surgeons in various ways to improve patient outcomes. While presenting a variety of exciting opportunities, the application of AI is not without limitations, making the adoption of this technology into clinical settings problematic. These challenges need to be addressed to ensure the safe and effective use of this technology.

## Data Availability

Not applicable.

## Abbreviations

AI: Artificial intelligence
ANN: Artificial Neural Network
AP: Anteroposterior
CE: *Conformité Européenne*
CNN: Convolutional Neural Network
CT: Computed Tomography
DAIR: Debridement, antibiotics, and implant retention
DL: Deep Learning
DNN: Deep Neural Network
FDA: US Food and Drug Administration
FDG-PET: Fluorodeoxyglucose-positron emission tomography
LOS: Length of stay in the hospital
MCID: Minimal clinically important difference
ML: Machine Learning
MRI: Magnetic resonance imaging
OA: Osteoarthritis
PJI: Prosthetic joint infection
PROMs: Patient-Reported Outcome Measures
PSI: Pelvic sagittal inclination
THA: Total hip arthroplasty
TKA: Total knee arthroplasty
